# Supplementary material for: Anthracycline-Induced Subclinical Right Ventricular Dysfunction in Breast Cancer Patients: A Systematic Review and Meta-Analysis
Source: Cancers (Basel). 2024 Nov 20;16(22):3883. doi: 10.3390/cancers16223883 (PMC11592457; doi:10.3390/cancers16223883)
Supplement: Supplementary file 1 [file cancers-16-03883-s001.zip › cancers-3276740-supplementary.pdf]

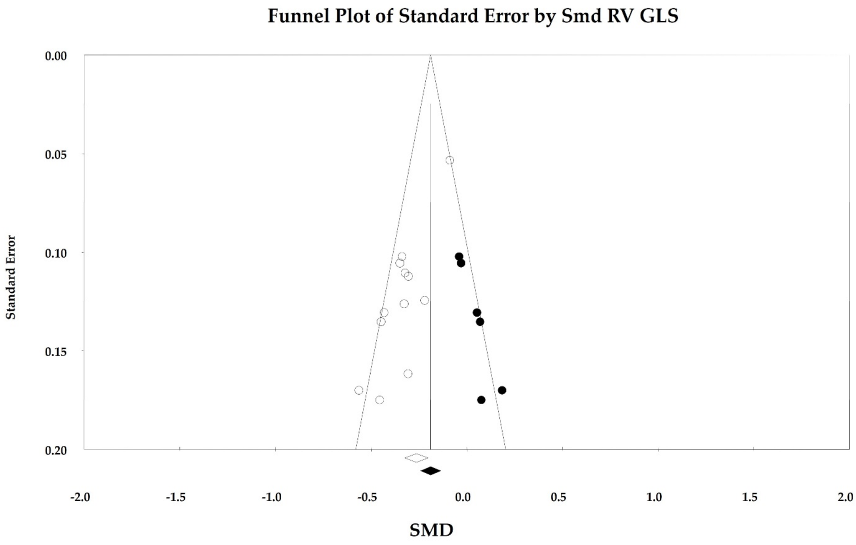

Figure S1. Funnel plot of standard error by standard means difference (SMD) of right vetricular global longitudinal strain (RV GLS).

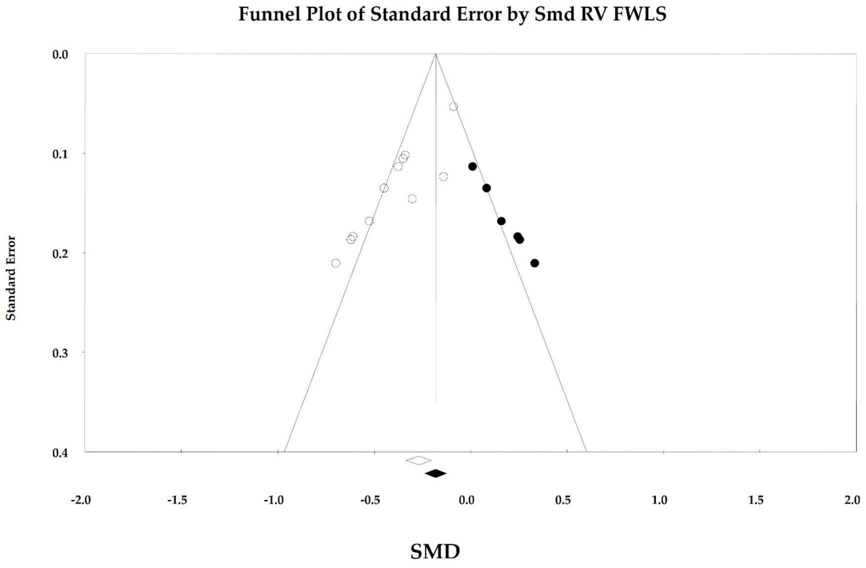

Figure S2. Funnel plot of standard error by standard means difference (SMD) of right vetricular free-wall longitudinal strain (RV FWLS).

Supplementary Table S1. Summary of Chemotherapy Regimens of the Studies Included in the Systematic Review and Meta-Analysis. ANT: Anthracycline, EPI: Epirubicin, DOX: Doxorubicin, PIRA: Pirarubicin, N.A.: not-available.

Author, Publication year	ANT drug name	Treatment duration	Number of cycles	Cumulative DOX equivalent dosage
Boczar, 2016 [29]	DOX or EPI	≅ 4 months	3–4	193.1 mg/m2
Chang, 2016 [30]	Probably EPI	≅ 2 months	3	212.1 mg/m2
Arciniegas Calle, 2018 [31]	DOX or EPI	≅ 5 months	2	252 mg/m2
Wang, 2018 [32]	PIRA	≅ 6 months	6	N.A
Anqi,	Probably EPI	≅ 6 months	6	217.9 mg/m2

2019 [33]				
Wang, 2020 [34]	PIRA	N.A	N.A.	N.A.
Xu, 2021 [35]	EPI	≅ 3 months	4	215.6 mg/m2
Attar, 2022 [36]	Probably EPI	≅ 6 months	N.A.	258.4 mg/m2
Laufer-Perl, 2022 [37]	DOX	≅ 3 months	N.A	238.5 mg/m2
El-Sherbeny, 2023 [38]	Probably EPI	≅ 3 months	4	219.6 mg/m2
Ghaznawie, 2023 [39]	DOX or EPI	N.A	N.A.	N.A
Giang, 2023 [40]	DOX	≅ 3 months	4	236 mg/m2
Fawzy, 2024 [41]	DOX or EPI	≅ 3-4 months	3–4	239.3 mg/m2
Gorgiladze, 2024 [42]	DOX or EPI	≅ 4 months	4	239.8 mg/m2
Rossetto, 2024 [43]	DOX	≅ 3-4 months	4–6	250 mg/m2

Supplementary Table S2. Newcastle-Ottawa scale (NOS) quality assessment form for nonrandomized studies included in the review. (*) = poor (0–4*), fair (5–6*), or good (>6*).

	**Selection**					**Comparability**		**Outcome**			**Overall Score**
**Study & Year**	**1**	**2**	**3**	**4**	**5**	**6**	**7**	**8**	**9**	**10**	**(*)**
Boczar, 2016 [29]	*	*	*	*	*		*	*	*	*	**9**
Chang, 2016 [30]	*		*	*	*		*	*	*		**7**
Arciniegas Calle, 2018 [31]	*		*	*	*		*	*	*		**7**
Wang, 2018 [32]	*		*		*		*	*	*		**6**
Anqi, 2019 [33]	*		*	*	*		*	*	*	*	**8**
Wang, 2020 [34]	*	*	*	*	*		*	*	*		**8**
Xu, 2021 [35]	*		*	*	*		*	*	*		**7**
Attar, 2022 [36]	*	*	*	*	*		*	*	*	*	**9**
Laufer-Perl, 2022 [37]	*		*	*	*		*	*	*	*	**8**
El-Sherbeny, 2023 [38]	*	*	*	*	*		*	*	*		**8**
Ghaznawie, 2023 [39]	*		*	*	*		*	*	*		**7**
Giang, 2023 [40]	*	*	*	*	*		*	*	*	*	**9**
Fawzy, 2024 [41]	*	*	*	*	*		*	*	*	*	**9**
Gorgiladze, 2024 [42]	*		*		*		*	*	*	*	**7**
Rossetto, 2024 [43]	*	*	*	*	*		*	*	*		**8**
